# What are the prospects for using complexes of copper(ii) and zinc(ii) to suppress the vital activity of *Mycolicibacterium smegmatis*?†

**DOI:** 10.1039/d1ra08555g

**Published:** 2022-02-10

**Authors:** Irina A. Lutsenko, Dmitry E. Baravikov, Kseniya A. Koshenskova, Mikhail A. Kiskin, Yulia V. Nelyubina, Petr V. Primakov, Yulia K. Voronina, Veronika V. Garaeva, Dmytry A. Aleshin, Teimur M. Aliev, Valery N. Danilenko, Olga B. Bekker, Igor L. Eremenko

**Affiliations:** N.S. Kurnakov Institute of General and Inorganic Chemistry of the Russian Academy of Sciences Leninsky prosp. 31, 119991 GSP-1 Moscow Russian Federation irinalu05@rambler.ru +7-495-952-1279; D.I. Mendeleev University of Chemical Technology of Russia M. Pirogovskaya str. 1a 119435 Moscow Russian Federation; A.N. Nesmeyanov Institute of Organoelement Compounds, Russian Academy of Sciences Vavilova str. 28 119991 Moscow Russian Federation; Vavilov Institute of General Genetics, Russian Academy of Sciences Gubkina 3119333 Moscow Russian Federation; Moscow Institute of Physics and Technology 9 Institutskiy per, Dolgoprudny Moscow Region 141701 Russian Federation

## Abstract

New complexes of zinc(ii) and copper(ii) with 2-furoic acid (Hfur), acetic acids and N-donor ligands with the compositions [Zn_2_(fur)_4_]_*n*_ (1), [Zn_2_(fur)_4_(NH_2_py)_2_] (2, NH_2_py = 3-aminopyridine), [Zn(fur)_2_(neoc)] (3, neoc = 2,9-dimethyl-1,10-phenantroline), [Zn(OAc)_2_(neoc)] (4, OAc = acetat-anion), and [Cu(fur)_2_(neoc)(H_2_O)] (5) were synthesized. The structures of the compounds were established by single crystal X-ray diffraction analysis. Complexes 1 and 2 are binuclear; whereas 3–5 are mononuclear. The stabilization of supramolecular architectures in crystals for compounds 1–5 occurs due to π–π-bonding between heterocycles and hydrogen interactions that provide good solubility in aqueous solutions. The stability of the complexes upon dissolution in 5% dextrose and 0.9% NaCl was confirmed by UV-vis spectroscopic and NMR (^1^H) data. The study of *in vitro* biological activity was carried out against the non-pathogenic strain of *Mycolicibacterium smegmatis* that is a model for *M. tuberculosis*. The synergistic effect of ligands is observed for complexes 3–5 and is characterized by an increase in the biological activity values. On passage from Zn^2+^ to Cu^2+^ complexes, the biological activity increases and the maximum effect is observed for compound [Cu(fur)_2_(phen)]. Analysis of the transcriptomic profiles of the *M. smegmatis mc*^*2*^*155* strain under the pressure of the copper complex [Cu(fur)_2_(phen)] made it possible to isolate 185 genes, one quarter of which are associated with the compensation of iron deficiency in the bacterial strain. Genes associated with the transport and metabolism of heavy metals, biosynthesis of fatty and amino acids, biodegradation and transport of urea were also isolated.

## Abstract 2

New complexes of zinc(ii) and copper(ii) with 2-furoic acid (Hfur), acetic acids and N-donor ligands with the compositions [Zn_2_(fur)_4_]*_n_*, [Zn_2_(fur)_4_(NH_2_py)_2_], [Zn(fur)_2_(neoc)], [Zn(OAc)_2_(neoc)], and [Cu(fur)_2_(neoc)(H_2_O)] were synthesized.

## Graphical abstract

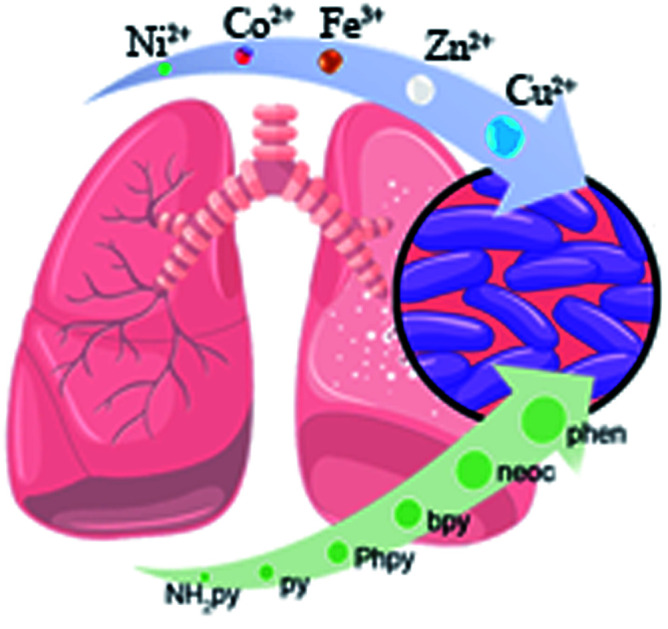

## Introduction

Tuberculosis is among the most hazardous diseases of mankind (according to the WHO) and takes the lives of tens of thousands of people every year.^1^ More than 130 years have passed since the discovery of the tuberculosis pathogen by R. Koch (1882) – Koch's bacillus – but doctors, biologists and biochemists still have not been able to definitively conquer this infection. The problem lies in the fast development of various types of resistance by mycobacteria which currently count more than a dozen, namely, drug resistance, multidrug resistance (MDR), HIV-associated varieties of tuberculosis, latent, dormant, multi-resistant and other species that cause global problems in the treatment of this disease.^2–6^ Creating drugs for the chemotherapy of tuberculosis is among the fundamental constituents of modern medicine. Obviously, the problem of searching for compounds with new mechanisms of action comes to the fore. Perhaps, coordination compounds with essential (vital) metals (Cu, Zn, Co, Fe, Mg *etc.*) might provide a solution. For example, this approach already proved efficient in the treatment of cancer, malaria, toxoplasmosis, and other hazardous diseases (cisplatin, auranofin, carboplatin, nitroprusside, silver sulfadiazine, *etc.*).^7–23^ Today there are no anti-tuberculosis drugs based on coordination compounds in medicine. Apparently, the development of such drugs is hindered by the insufficient understanding of the role of metal ions in the physiology and homeostasis of mycobacteria. Nevertheless, work in this direction is underway, as shown by an exponential increase in the number of publications proving the efficiency of complexes in suppressing the vital activity of mycobacteria and other pathogens.^24–29^ 2-Furoic acid (pyromucic acid, Hfur) was taken as the organic ligand to create complexes with anti-tuberculosis activity. The furan core of this compound is an ancestor of a group of antimicrobial drugs such as furazolidone, furadonin, enterofuril, *etc.* Earlier, S. Melnic *et al.*^30^ showed that heterometallic triangular {Fe_2_CoO} complexes with fur^−^ anions exhibited *in vitro* activity against *Mycobacterium tuberculosis* H37Rv. Of the compounds with the composition [Fe_2_MO(fur)_6_L] studied (M = Mn^2+^, Co^2+^, Ni^2+^; L = H_2_O, THF, 3Cl-Py), the [Fe_2_Co(O)(fur)_6_(THF)(H_2_O)_2_] heteronuclear complex was found to have *in vitro* biological activity against *M. tuberculosis* H37Rv. Further studies of furoate complexes with various cations (Ni^2+^, Fe^3+^, Co^2+/3+^, Zn^2+^, Cu^2+^) showed that the mycobacterium was most sensitive to Zn^2+^ and Cu^2+^.^30–35^ In continuation of the “structure activity relationship (SAR)” studies, Zn(ii) complexes were synthesized, namely, polymeric [Zn_2_(fur)_4_]_*n*_ (1), bi-[Zn_2_(fur)_4_(NH_2_py)_2_] (2, NH_2_py = 3-aminopyridine) and mononuclear [Zn(fur)_2_(neoc)] (3, neoc = 2,9-dimethyl-1,10-phenantroline), [Zn(OAc)_2_(neoc)] (4) and [Cu(fur)_2_(neoc)(H_2_O)] (5). The structures of all the compounds obtained were resolved by direct single-crystal X-ray diffraction analysis. The stability of complexes 1–5 was confirmed by UV-vis spectroscopy upon dissolution in physiological 0.9% NaCl and 5% glucose solutions. The stability of compound 3 was studied by the NMR (^1^H) method in a DMSO solution. The biological activity of 1–5*in vitro* was determined against non-pathogenic *Mycolicibacterium smegmatis* (a model for *M. tuberculosis* H37Rv).

## Experimental

### Preparation of complexes

Commercial reagents and solvents were used for the synthesis: 2-furoic acid (99%, “J&K”), Zn(AcO)_2_·2H_2_O (≥99%), Cu(AcO)_2_·2H_2_O (≥99%), ZnO (≥99%), neocuproine hemihydrate (≥99%, “Acros Organics”), 3-aminopyridine (≥99%, “Acros Organics”), acetonitrile (≥99%), ethanol (≥95%), NaCl (0.9%), dextrose (5%), MeOH, CHCl_3_, DMSO and distilled water.

#### Synthesis of [Zn_2_(fur)_4_]_*n*_ (1)

ZnO (0.08 g, 1 mmol), Hfur (0.230 g, 2.05 mmol), were dissolved in mixture MeCN : EtOH : H_2_O (6 : 1 : 1, respectively) at 70 °C, air and stirred until dissolution of ZnO. The final solution was filtered and concentrated. Crystals were obtained after 24 hours. Yield 0.46 g (80%). Anal. calc. Zn_2_O_12_C_20_H_12_: C 41.81, H 2.1. Found: C 41.42, H 2.4. FT-IR (ATR), *ν*/cm^−1^: 3140 v/w, 2104 v/w, 1733 v/w, 1634 m, 1583 s, 1558 s, 1543 s, 1473 *vs.*, 1419 *vs.*, 1373 *vs.*, 1230 m, 1205 s, 1140 w, 1078 w, 1010 m, 937 m, 884 m, 835 w, 799 m, 777 s, 754 *vs.*, 612 m, 593 m, 577 w, 515 s, 487 m, 451 v/w, 431 v/w, 415 v/w, 407 v/w.

#### Synthesis of complex [Zn_2_(fur)_4_(NH_2_py)_2_] (2)

Zn(AcO)_2_·2H_2_O (0.220 g, 1 mmol), Hfur (0.230 g, 2.05 mmol) and 3-NH_2_py (0.100 g, 1 mmol), were dissolved in 20 ml MeCN (70 °C, air) and stirred for one and a half hour. The final solution was filtered and concentrated. Crystals were obtained after 24 hours. Yield 0.39 g (61%). Anal. calc. Zn_2_O_12_C_30_H_24_N_4_: C 47.21, H 3.17, N 7.34. Found: C 47.23, H 3.21, N 7.38. FT-IR (ATR), *ν*/cm^−1^: 3483 v/w, 3389 w, 3226 v/w, 3145 v/w, 3123 v/w, 3058 v/w, 2167 v/w, 2148 v/w, 1712 v/w, 1640 *vs.*, 1614 s, 1577 *vs.*, 1481 *vs.*, 1454 m, 1412 *vs.*, 1395 *vs.*, 1368 *vs.*, 1310 m, 1273 m, 1225 m, 1195 s, 1140 m, 1077 m, 1063 m, 1017 m, 1007 m, 935 m, 900 w, 883 m, 861 w, 800 s, 781 *vs.*, 756 *vs.*, 658 m, 696 s, 615 m, 597 m, 546 w, 484 s.

#### Synthesis of [Zn(fur)_2_(neoc)] (3)

Zn(AcO)_2_·2H_2_O (0.220 g, 1 mmol), Hfur (0.230 g, 2.05 mmol) and neoc (0.210 g, 1 mmol), were dissolved in 20 ml MeCN (70 °C, air) and stirred for one and a half hour. The final solution was filtered and concentrated. Crystals were obtained after 24 hours. Yield 0.32 g (70%). Anal. calc. ZnO_6_C_24_H_18_N_2_: C 58.18, H 3.63, N 5.66. Found: C 58.31, H 3.63, N 5.72. FT-IR (ATR), *ν*/cm^−1^: 3121 v/w, 3071 v/w, 2159 v/w, 1761 v/w, 1620 m, 1559 m, 1520 w, 1474 s, 1394 s, 1353 *vs.*, 1297 m, 1228 m, 1193 s, 1164 w, 1135 m, 1108 m, 1075 m, 1011 s, 932 m, 883 m, 864 m, 845 m, 805 m, 771 *vs.*, 751 s, 728 s, 681 m, 661 m, 617 m, 598 m, 569 m, 549 m, 474 s. ^1^H NMR (300 MHz, DMSO, 294 K): 3.09 (s, 6H, Me-neoc), 6.52 (dd, 2H, 4-fur, ^3^*J*_H–H_ = 3.2 Hz, ^3^*J*_H–H_ = 2.2 Hz), 6.96 (d, 2H, 3-fur, ^3^*J*_H–H_ = 3.2 Hz), 7.72 (brs, 2H, 5-fur), 8.05 (d, 2H, 3/8-neoc, ^3^*J*_H–H_ = 8.4 Hz), 8.20 (s, 2H, 5/6-neoc), 8.83 (d, 2H, 4/7-neoc, ^3^*J*_H–H_ = 8.4 Hz). Mixture of ligands: ^1^H NMR (300 MHz, DMSO, 294 K): 2.79 (s, 6H, Me-neoc), 6.65 (dd, 1H, 4-fur, ^3^*J*_H–H_ = 3.4 Hz, ^3^*J*_H–H_ = 2.0 Hz), 7.20 (d, 1H, 3-fur, ^3^*J*_H–H_ = 3.4 Hz), 7.61 (d, 2H, 3/8-neoc, ^3^*J*_H–H_ = 8.2 Hz), 7.86 (s, 2H, 5/6-neoc), 7.91(brs, 1H, 5-fur), 8.34 (d, 2H, 4/7-neoc, ^3^*J*_H–H_ = 8.2 Hz), 13.09 (brs, 1H, 2-fur).

#### Synthesis of [Zn(AcO)_2_(neoc)] (4)

Zn(AcO)_2_·2H_2_O (0.220 g, 1 mmol) and neoc (0.210 g, 1 mmol), were dissolved in 20 ml MeCN (70 °C, air) and stirred for one and a half hour. The final solution was filtered and concentrated. Crystals were obtained after 24 hours. Yield 0.27 g (70%). Anal. calc. ZnO_4_C_18_H_18_N_2_: C 55.24, H 4.6, N 7.16. Found: C 55.31, H 4.65, N 7.20. FT-IR (ATR), *ν*/cm^−1^: 3052 v/w, 2997 v/w, 2927 v/w, 1616 s, 1590 s, 1567 s, 1503 m, 1421 s, 1378 *vs.*, 1327 s, 1226 w, 1158 w, 1009 m, 924 brw, 867 s, 813 w, 779 w, 732 w, 677 *vs.*, 618 m, 549 m, 501 w, 434 m, 405 m.

#### Synthesis of [Cu(fur)_2_(neoc) H_2_O] (5)

Cu(AcO)_2_·2H_2_O (0.220 g, 1 mmol), Hfur (0.224 g, 2 mmol) and neoc (0.208 g, 1 mmol) were dissolved in mixture MeCN : CHCl_3_ : MeOH (in a ratio of 4 : 2 : 1, respectively; 60 °C, air) and stirred for one and a three hours. The final yellow-green solution was filtered and concentrated. Green crystals were obtained after 24 hours. Yield 0.42 g (82%). Anal. calc. CuO_7_C_24_H_20_N_2_: C 56.30, H 3.94, N 5.47. Found: C 56.38, H 3.89, N 5.48. FT-IR (ATR), *ν*/cm^−1^: 3119 w, 1596 s, 1548 m, 1474 s, 1395 s, 1353 *vs.*, 1223 m, 1189 s, 1134 m, 1074 m, 1012 m, 931 m, 862 m, 808 s, 772 *vs.*, 726 s, 597 m, 542 s, 470 s, 418 m.

### IR spectra and CHN

IR spectra were recorded in the 400–4000 cm^−1^ region using a Spectrum-65 PerkinElmer FT-IR spectrometer. Microprobe analyses were carried out using an Carlo Erba EA 1108 Series CHN Elemental Analyser (Center of Collective Use of IGIC RAS).

### NMR spectra

^1^H and DOSY NMR spectra were recorded from DMSO solutions with a Bruker Avance 300 FT-NMR spectrometers (^1^H frequency: 300.13 MHz) equipped with 5 mm probe with Z-gradient. All spectra were referenced to the chemical shift of a deuterated solvent (^1^H 2.50 ppm). The 2D DOSY spectra were obtained using a bipolar gradient pulse sequence (LEDbpgp2s) with pre-equilibrated for 10 minutes inside spectrometer at 305 K and processed with Bruker 2.1 TOPSPIN. Parameters of 90° angle and gradient pulses were determined manually (see details in ESI†). Two samples were prepared for the taking ^1^H NMR and DOSY spectra. The first one with the complex 3 and the second one with ligands mixture, which were taken with the stoichiometric ratio neoc : Hfur = 1 : 2.

### X-ray crystallography

X-ray diffraction experiments for 1, 3–5 were carried out at 120 K with a Bruker APEX2 DUO CCD diffractometer, those for 2 at 150 K with a Bruker AXS Smart Apex II CCD diffractometer, both using graphite monochromated Mo-Kα radiation (*l* = 0.71073 Å). Using Olex2,^36^ the structures were solved with the ShelXT^37^ structure solution program using Intrinsic Phasing and refined with the olex2.refine^38^ refinement package using Gauss–Newton minimization against *F*^2^ in anisotropic approximation for non-hydrogen atoms. Hydrogen atoms of NH groups in 2 were located from difference Fourier synthesis; positions of other hydrogen atoms were calculated, and they all were refined in isotropic approximation within the riding model. Crystal data and structure refinement parameters for 1–5 are given in Table S1. CCDC 2112367 (1), 2112368 (2), 2112369 (3), 2112370 (4) and 2115090 (5) contain the supplementary crystallographic data for this paper (Table S1†).

### Spectroscopy and stability

The UV-vis spectra were obtained using Shimadzu UV-2600 spectrophotometer in SoloPharm 0.9% NaCl and SoloPharm 5% glucose solutions in the range of 220–400 nm. The stability of the complexes in solution was monitored by measuring the spectra of the sample (50 μM) once every 2 hours for 48 hours at room temperature.

### Antibacterial activity

To determine the biological activity of substances 1–5 possessing anti-tuberculosis properties in the *M. smegmatis mc*^*2*^*155* test system, the paper disk method was used. The technique involved determining the size of the zone of inhibition of the growth of the strain seeded as a lawn on an agar medium, around paper disks containing the compound in various concentrations. The bacteria washed off Petri dishes with tryptone soya agar M-290 medium (Himedia) were grown overnight in Lemco-TW liquid medium (Lab Lemco' Powder 5 g L^−1^ (Oxoid), peptone special 5 g L^−1^ (Oxoid), NaCl 5 g L^−1^, Tween-80) at +37 °C until the average logarithmic growth phase at optical density OD600 = 1.5, then mixed with molten agar medium M-290 in a ratio of 1 : 9 : 10 (culture : Lemco-TW : M-290) and the resulting mixture was poured as a top layer onto Petri dishes, 5 ml per dish, with 20 ml already solidified M-290 agar medium. After the agar in the top layer solidified, paper disks soaked with a solution of the test substance were placed on the plate surface. The culture was incubated for 24 hours at +37 °C. The diameter of the zone of inhibition of *M. smegmatis mc*^*2*^*155* growth around the paper disk impregnated with the compound was determined. The MIC (minimum inhibiting concentration) was taken as the concentration of the compound where the zone of growth inhibition was the smallest.

### Transcriptome data analysis (RNA-seq)

Total RNA extracted from six independent samples (three control replicates – untreated cultures, and three experimental replicates – treated with an cooper complex) subjected to RNA sequencing. *M. smegmatis* cultures were grown in Middlebrook 7H9 medium (Himedia, India) supplemented with 0.05% (v/v) Tween 80 and 0.5% (v/v) glycerol at 37 °C and 250 rpm before OD600 = 1,2 (log-phase of growth). Then copper complex was added to experimental culture at a concentration of ¼ MIC (MIC was previously determined in liquid medium), and the culture was incubated at 37 °C and 250 rpm for 90 minutes. Untreated culture was incubated under the same conditions for 90 minutes. Cells from 30 ml culture were harvested by centrifugation for 10 min at 4000×*g* and 4 °C, washed by 40 ml of fresh Middlebrook 7H9 broth. Total RNA was extracted as described by Rustad *et al.*^39^*M. smegmatis* cells were homogenized in extractRNA reagent Trizol (Invitrogen, USA), followed by phenol (pH = 4.5)–chloroform/isoamyl alcohol (25 : 24 : 1) purification, precipitation with isopropanol (2 : 1, v/v) and washed with 80% ethanol. Remaining genomic DNA was removed by DNAse I (Invitrogen, USA). Ribosomal RNA was removed using the Ribo-Zero Plus rRNA Depletion Kit (Illumina) and libraries were prepared using the NEBNext® Ultra II Directional RNA Library Prep Kit (NEB). Libraries were subsequently quantified by Quant-iT DNA Assay Kit, High Sensitivity (Thermo Fisher Scientific). Equimolar quantities of libraries were sequenced by a high throughput run on the Illumina HiSeq using 2 × 100 bp paired-end reads and a 1% phiX-in control. Raw reads of the *M. smegmatis mc*^*2*^*155* transcriptome with the complex lie in the NCBI database (Bioproject https://www.ncbi.nlm.nih.gov/bioproject/PRJNA747161). Raw reads' quality was assessed by FASTQC v0.11.9.^40^ The remaining adapters were removed with Trimmomatic v0.39.^41^ Reads were mapped to the *M. smegmatis mc*^*2*^*155* reference assembly (CP000480.1) and quantified with HISAT2 v.2.2.1.^42^ Differential expression analysis was performed using edgeR v3.30.3 package.^43^

## Results and discussion

### Synthesis of complexes 1–5

**Scheme 1:**
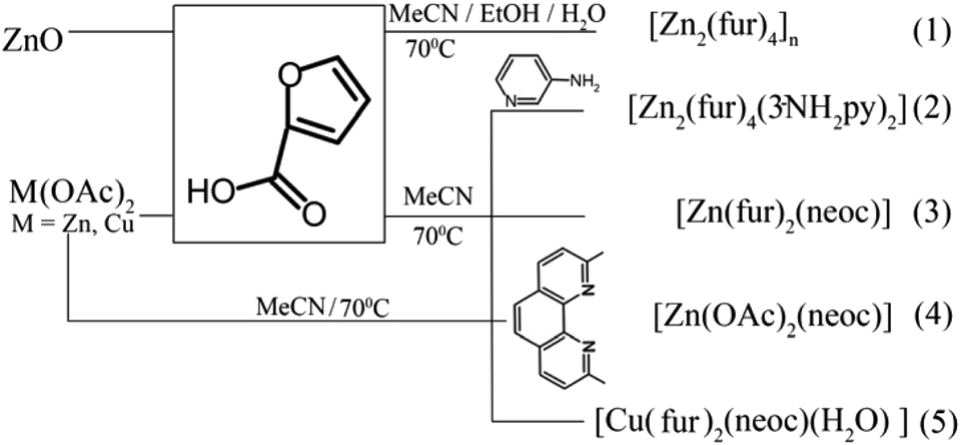
Synthetic routes to compounds 1–5.

A small nuclearity of the complex should most likely be one of the main criteria for the would-be coordination-based chemotherapy drugs against tuberculosis (by analogy with the Lipinski's rule that imposes certain restrictions on hypothetical organic pharmaceuticals). Scheme 1 shows the synthesis routes for complexes 1–5. In contrast to 2–5, polymer complex 1 was obtained from the oxide, whereas the adducts with N-donor ligands for 2–5 were synthesized from acetates by anion exchange reactions.

### Single crystal X-ray structure of 1–5

**Fig. 1:**
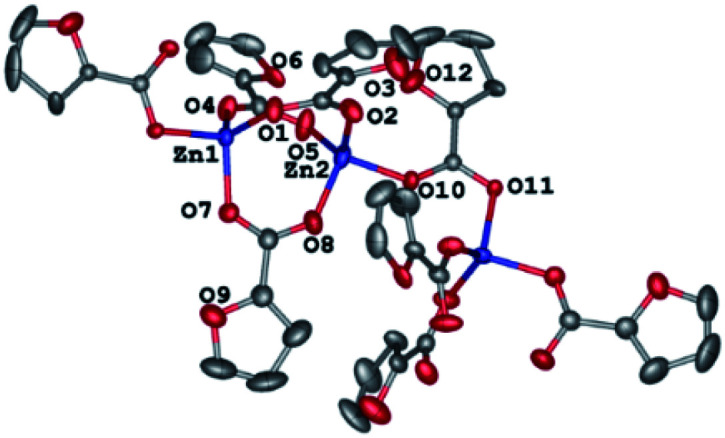
A fragment of a 1D-coordination polymer chain in the crystal of 1. Hereinafter, hydrogen atoms H(C) and minor components of the disordered anions are omitted for clarity, non-hydrogen atoms are shown as thermal ellipsoids at 50% probability level, and labels are given for symmetry-independent heteroatoms only.

As identified by X-ray diffraction, the compound 1 (Fig. 1) is a 1D-coordination polymer with two symmetry-independent zinc(ii) ions and four fur^−^ anions. Each metal ion coordinates four anions (Table 1) that form its distorted tetrahedral coordination environment.

**Table 1:** Selected distances (Å) for 1–5

	Compounds				
	1	2	3	4	5
M–O (fur^−^, AcO^−^)	1.925(2), 1.9672(18)	1.969(4), 2.135(1)	1.9485(2), 1.976(2)	1.911(3), 2.43(3)	1.947(2), 1.981(2)
M–N	—	2.0237(14)	2.057(2), 2.070(2)	2.082(3), 2.102(3)	2.020(4), 2.254(4)
Zn⋯Zn	3.3066(5)	3.0193(3)	—	—	—

**Fig. 2:**
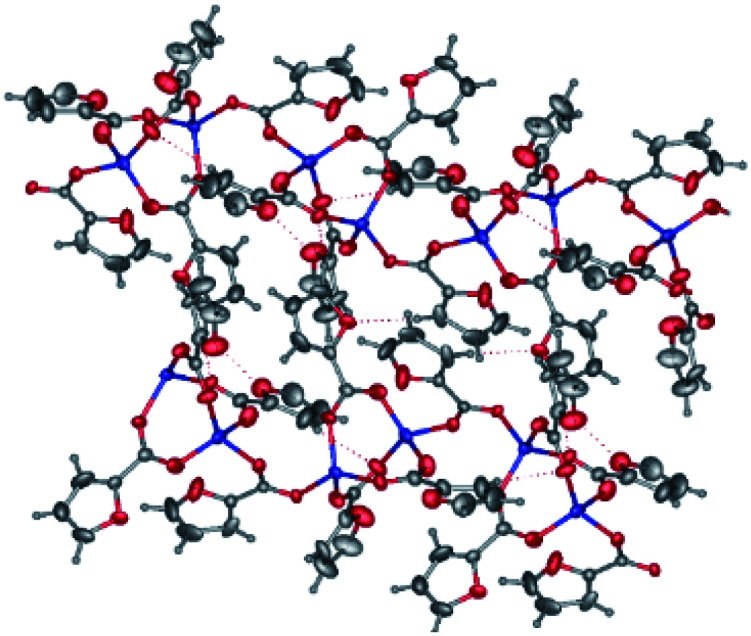
Formation of a 1D polymer chain for 1.

Carboxylate groups of the three bridging anions bind these two zinc(ii) ions to produce binuclear nodes (Zn–O 1.925(2)–1.966(2) Å, Zn⋯Zn 3.3066(5) Å) that are connected to each other by one carboxylate group (Zn–O 1.9293(19), 1.9672(18) Å, Zn⋯Zn 4.4320(5) Å) to produce zigzag 1D-polymer chains as sometimes found in other 1D-coordination polymers based on transition metals and carboxylate-derived ligands (see, *e.g.*^44,45^). In the crystal of 1, these chains run along the crystallographic axis *b* with the separation Zn(1)⋯Zn(2) and an angle Zn(1)Zn(2)Zn(1) are 3.306(2) Å and 116.60(1)–145.84(1)°, respectively. They are held together only by weak interactions, such as C–H⋯O, π⋯π and H⋯H contacts (Fig. 2).

**Fig. 3:**
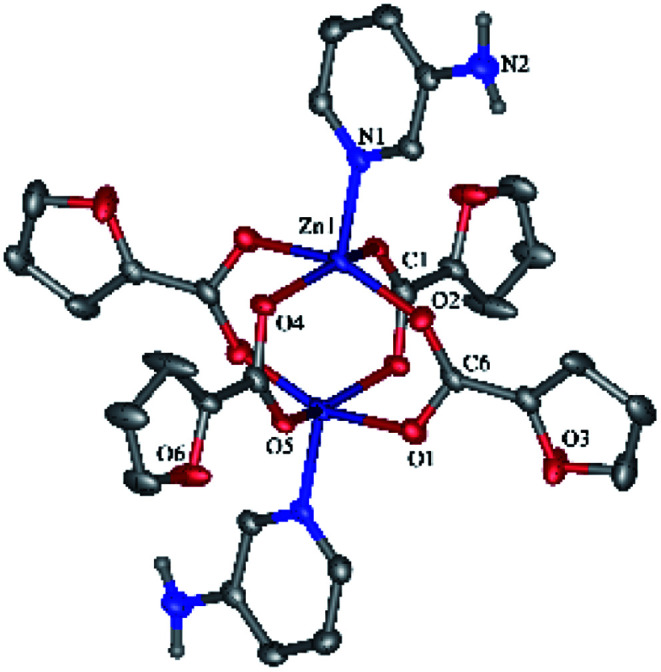
General view of 2 in representation of atoms as thermal ellipsoids at 50% probability level. Hydrogen atoms except those of NH groups and minor components of the disordered anions are omitted for clarity and labels are shown for symmetry-independent heteroatoms only.

Molecules of binuclear zinc complex 2 in the crystal are in a special position at the crystallographic inversion center. Inversion center is located between the two Zn atoms. Zinc ions in 2 have a square pyramid (*τ* = 0.008, O1, O2, O4 and O5 atoms lie at the base of the pyramid) formed by four bridging fur^−^ anions and one monodentate aminopyridine ligand (CN = 5) (Fig. 3). The distances Zn–O, Zn–N and Zn⋯Zn, are given in Table 1.

The molecule consists of two almost perpendicular flat fragments (the angle between the planes Zn1–N1–C1 and Zn1–C6–Zn1 is 86.93°) with some deviation of the oxygen atoms of the carboxyl groups and the aromatic fragments. Thus, the fur^−^ and aminopyridine fragments are deployed relative to the Zn1–N1–C1 plane at 9.63° and 19.13° respectively, and the furan cycle is deployed relative to the Zn1–C6–Zn1 plane at 18.19°. The molecular packing in a crystal consists of infinite layers parallel to the *a0b* plane, formed by the classical NH⋯O hydrogen bonds and additionally stabilized by π⋯π interactions of aromatic fragments (the distances between the centroids of the rings are 3.4–3.9 Å, between the planes 3.5–3.6 Å). The layers are linked by CH⋯O interactions (Table 3).

**Fig. 4:**
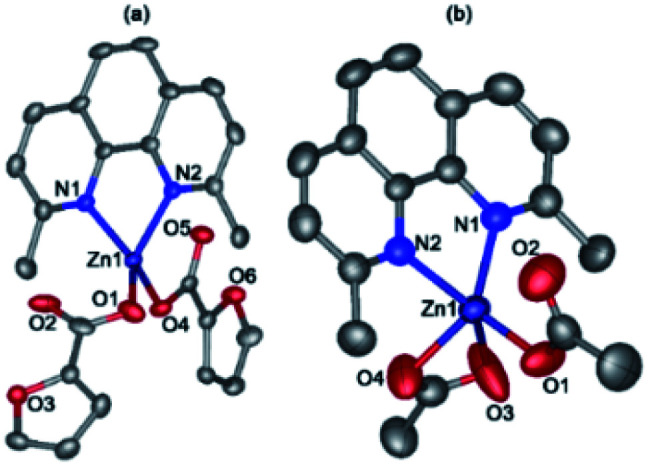
Molecular structures of 3 (a) and 4 (b).

Compounds 3 (Fig. 4a) and 4 (Fig. 4b) have a very similar molecular structures (Table 1) with the zinc ion binding one neoc ligand (Zn–N 2.057(2)–2.102(3) Å) and two fur^−^ or Ac^−^ anions (Zn–O 1.9485(18)–1.976(2) and 1.911(3)–2.43(3) Å) that coordinate it in a monodentate mode or in monodentate and chelate modes, respectively, as often found in other transition metal complexes with phenanthroline- and carboxylate-derived ligands (see, *e.g.*).^46^ The resulting coordination environment of the zinc(ii) ions in 3 is a distorted tetrahedron while in 4, it is better described as a square pyramid (*τ* = 0.18; O1, O4, N1 and N2 atoms lie at the base of the pyramid).

**Fig. 5:**
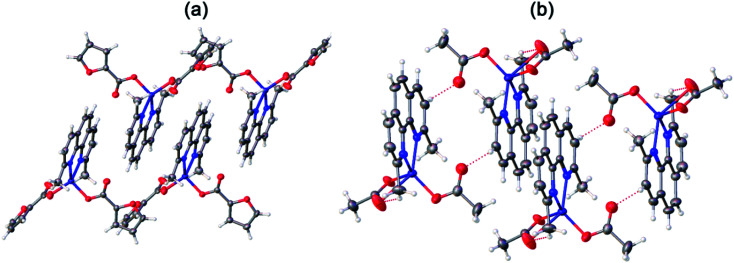
General view of a fragments crystals packing 3 (a) and 4 (b) illustrating the formation of infinite chains by stacking interactions between the neoc ligands.

In both crystals, the aromatic neoc ligands form parallel-displaced stacking interactions with intercentroid and shift distances of 3.566(2)–3.887(2) and 1.074(2)–1.762(2) Å and an angle between the planes of 0.0(5)–1.94(2)°. They, however, pack the molecules of these complexes into two different types of supramolecular associates, the infinite chains in 3 (Fig. 5a) and centrosymmetric dimers in 4 (Fig. 5b); the latter are held together by weak intermolecular C–H⋯O contacts to produce similar infinite chains along the crystallographic axis *a*.

**Fig. 6:**
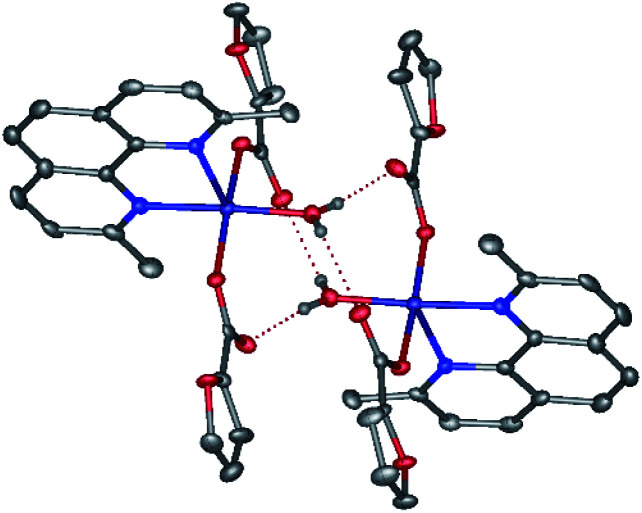
A fragment of crystals packing 5 illustrating the formation of centrosymmetric dimers by O–H⋯O hydrogen bonds.

In contrast, the metal ion in the discrete copper(ii) complex 5 (Fig. 6, Table 2) coordinates one neoc ligand in a bidentate mode (Cu–N 2.020(4), 2.254(4) Å), two fur^−^ anions (Cu–O 1.954(2), 1.981(2) Å) and an additional water molecule (Cu–O 1.981(2) Å) to produce a distorted square pyramid coordination environment (CN = 5; *τ* = 0.07; O1, O4, O10 N2 atoms form the base of the pyramid).

**Table 2:** H-bonds in crystal of 2 and 5

H-bond	D–H, Å	H⋯A, Å	D⋯A, Å	D–H⋯A, °
2				
N2–H2A⋯O6	0.91	2.32	3.175(11)	156
N2–H2A⋯O6′	0.91	2.29	3.110(13)	149
N2–H2B⋯O1	0.87	2.53	3.169(7)	131
C5′–H5′⋯O4′	0.95	2.39	3.214(11)	145
C8–H8⋯O3	0.95	2.45	3.32(2)	153
C13–H13⋯O5′	0.95	2.51	3.324(17)	144
C15–H15⋯O1	0.95	2.56	3.180(15)	123

5				
O10–H10^b^⋯O5	0.87	1.84	2.638(4)	123
O10–H10^a^⋯O2	0.85	2.07	2.695(3)	167

The molecules of the complex are assembled into centrosymmetric dimers by hydrogen bonds (O⋯O 2.638(4)–2.695(3) Å, OHO 123.7(3)–167(5)°) between the above water molecule and oxygen atoms of the anions that are not involved in the coordination to the copper(ii) ion. Despite the presence of the aromatic neoc ligand, no stacking interactions are observed for 5.

### UV-vis spectroscopy studies

**Fig. 7:**
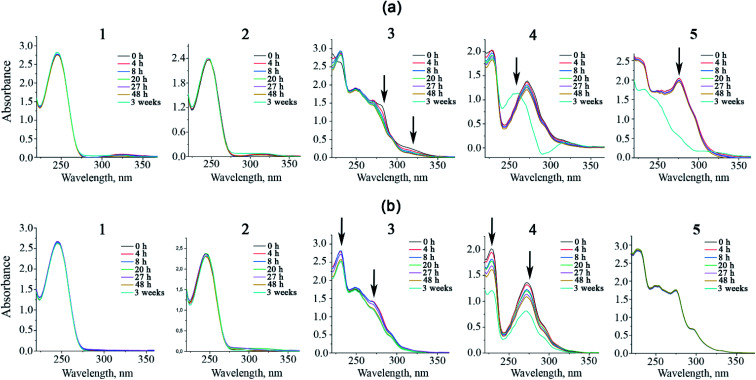
UV-vis spectra in 5% dextrose (a) and 0.9% NaCl (b) at room temperature.

The electronic absorption spectra of 1–5 (5 × 10^−5^ M) were collected in saline (0.9% NaCl) and 5% dextrose containing saline for 48 hours at room temperature (Fig. 7a and b). Additionally, the investigated samples were stored for extra 3 weeks to check its long-term stability in the given conditions. All complexes exhibit high absorption rates in the high-energy region. For compounds 1 and 2, the absorption band at 250 nm is conditioned by effective intraligand π–π* and n–π* transitions within the fur^−^ ligands.^47^ Complexes 3–5 show the additional red-shifted bands at about 275 nm correspond to intraligand transitions within coordinated neocuproine. For all complexes, low intensity absorption bands lying in the region of 300–350 nm are attributed to metal-to-ligand charge-transfer (MLCT).^48,49^ Saline solutions of 1, 2 and 5 are stable at room temperature for 48 hours, as no significant changes in the absorbance were noticed. However, prolonged storage of aforementioned species resulted in general decreasing of absorbance because of complex aggregation processes.^50^ Dextrose containing solutions of 1 and 2 have similar short term stability, but upon the storage for over 3 weeks, a new broad low intensity absorption band appears at 275–300 nm region, probably caused by anion exchange and overall partial hydrolysis of the species (Fig. 7a).^51^ Both saline and dextrose solutions of 3 and 4 show similar absorption intensity decrease within first 48 hours. Such spectral changes indicate that upon given conditions the complexes are slowly hydrolyzing. Nevertheless, the hydrolysis rate of both complexes in saline solutions are rather low, as the process is still going even after 3 weeks of storage. Complex 4 and 5 seems to be less stable in dextrose solution. After 3 weeks of storage, a completely new blue-shifted absorption band assigned to a different complex stoichiometry appeared.

### NMR (^1^H)

**Fig. 8:**
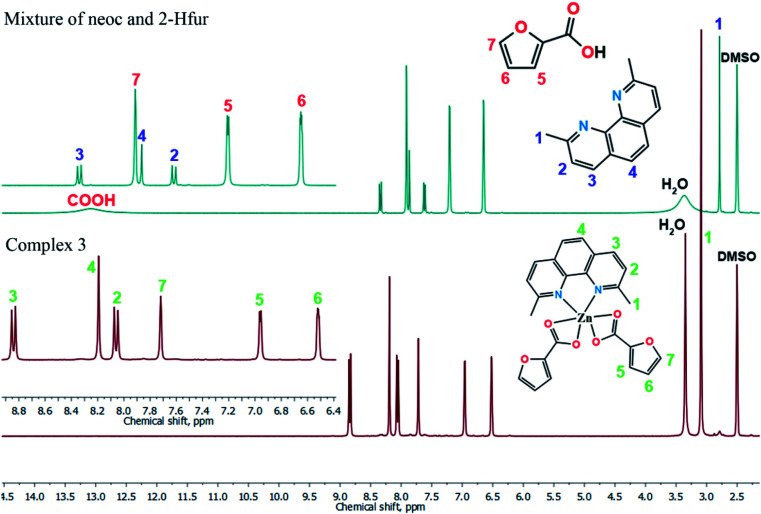
The comparison of a mixture and complex 3 (^1^H) NMR spectra.

The formation of donor–acceptor bond between a metal ion and a ligand leads to a redistribution of electron density distribution of the latter. As a result, it affects to chemical shift values of ligand's nuclei.^52,53^ Experimental ^1^H NMR spectra show that signals of all protons are shifted in the spectrum of the complex in comparison with the spectrum of ligands mixture (Tables S2 and S3;†Fig. 8), which proves the stability of the complex 3 in solution. The full assignment of protons for the ligands mixture and complex 3 samples presented in experimental section.

#### Diffusion

In contrast to DOSY spectrum of complex 3, the spectrum of ligands mixture clearly indicates two different self-diffusion coefficients for neoc and Hfur. Also, self-diffusion coefficient is inversely related to the molecule size, so for complex dissuasion must vary significantly from ligands coefficients that also proved by experimental values of diffusion coefficient (see ESI 2†). The quantitative relation between size of molecule and diffusion coefficient is possible to determine with the Stokes–Einstein eqn (1) which relating the self-diffusion coefficient (*D*) at a constant temperature (*T*) with the viscosity of solution (*η*) and hydrodynamic radius of a spherical particle (*r*).1
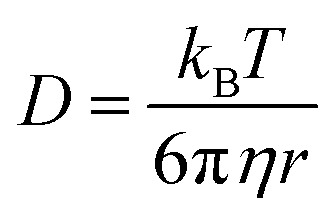


The Stokes–Einstein equation implies the relation between diffusion coefficient and radius of molecule for the spherical particles. However, the molecular shape of particles is not necessarily spherical. In general, the ratio for two different particles with diffusion coefficients *D*_*j*_, *D*_*i*_ in same viscosity samples is inverse proportional to the third-degree root of their molecular weights ratio for spherical particles and to the root for elongated spheroids.^53^ In this way the ration of self-diffusion coefficients for two different species should be in the range represented in expression 2.2
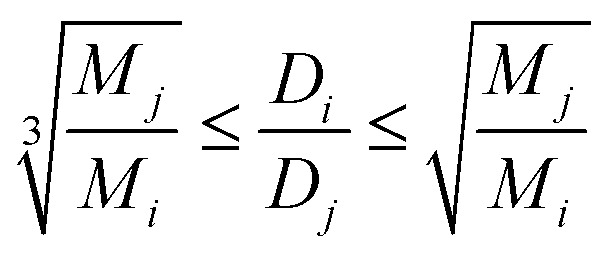
where *D*_*i*_, *D*_*j*_ – experimental values of self-diffusion coefficients for *i*th and *j*th particles in solutions with equals viscosity. *M*_*i*_, *M*_*j*_ – molecular weights of these species.

The experimental diffusion coefficients were obtained for two different samples described in experimental section. Thus, we need know viscosities of two samples to determine self-diffusion coefficients but as mentioned in ref. 54 the ration of self-diffusion coefficients of a molecule and a reference compound is independent of viscosity of solution. Hence, using DMSO as internal standard for two samples we can obtain the “true” ration of self-diffusion coefficient of the complex and ligands. The expression 3 defining viscosity-independent ratio for neocuproine and complex 3 self-diffusion coefficients is 1.34 that in good agreement with spherical particles approximation (1.34) according to eqn (2) and proves the stability of complex in a solution. All experimental self-diffusion coefficients and DOSY spectra can be found in ESI.†3
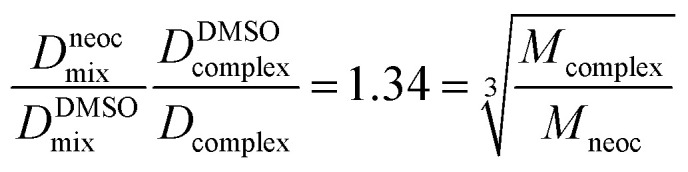
where *D*^DMSO^_mix_, *D*^DMSO^_complex_ – experimental values of DMSO self-diffusion coefficients in the mixture and complex samples. *D*^neoc^_mix_, *D*_complex_ – experimental values of neocuproine and complex 3 self-diffusion coefficients in the mixture and complex samples.

### Antibacterial activity

**Scheme 2:**
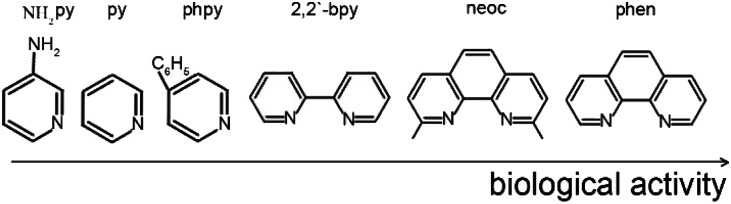
A number of activities of N-donor ligands in relation to *M. smegmatis*.

The antibacterial activity of compounds 1–5, was determined *in vitro* against a non-pathogenic *M. smegmatis* strain. It is known that the resistance of mycobacteria to chemotherapeutic agents is due to the low permeability of the mycobacterial cell wall that has an unusual structure. *M. smegmatis* are fast-growing non-pathogenic bacteria and are therefore used as organisms simulating the slow-growing *M. tuberculosis* bacteria, as well as for primary screening of anti-tuberculosis drugs.^55^ The *M. smegmatis* test system exhibits a higher degree of resistance to antibiotics and antituberculosis agents than *M. tuberculosis*, therefore the selection criterion is a compound concentration of <100 μg per disc., in contrast to *M. tuberculosis* (MIC <2 μg ml^−1^).^56^ All the results obtained for *in vitro* bioactivity of the compounds studied were compared to the activity of isoniazid (INH) and rifampicin (Rif), *i.e.*, the first line drugs for tuberculosis treatment under these experimental conditions. The concentration of the compound at which the minimum visible zone of growth inhibition is observed is considered as the MIC (minimum inhibitory concentration, μg per disc.). The biological activity of individual Hfur against *M. smegmatis* is comparable to that of INH (Table 3) but this effect persists for only one day. The results of antibacterial activity in the *M. smegmatis mc*^*2*^*155* test system and its variation over time for compounds 1–5 are shown in Table 3. Polymer complex 1 shows the smallest activity (534 μg per disk) among the compounds obtained (Table 3). Indeed, a considerable body of experimental data shows that in the absence of additional ligands (for example, N-donor ones), furancarboxylate complexes of Cu^II^, Zn^II^, Co^II^, and Fe^III^ exhibit low *in vitro* biological activity with respect to *M. smegmatis*.^57–59^ Moreover, it was noted that pyridine, aminopyridine, and phenylpyridine (in the case of copper complexes) could hinder the biological efficiency of compounds with respect to the strain.^31–34^ In contrast, 1,10-phenanthroline and its methylated derivative, neoc, show a synergistic effect: they increase bioactivity by almost an order of magnitude (534 μg per disk for 1 and 50 for 3). If the fur anion is replaced by Ac^−^, the biological activity of 4 towards *M. smegmatis* increases more than twofold (21 μg per disk). If Cu^2+^ is used as a complexing agent, the biological activity increases to 12 μg per disk for 5 (however, it remains lower than that of the previously obtained complex with 1,10-phenantroline [Cu(fur)_2_(phen)^31^) that shows the best result (Table 3). Thus, the N-donor ligands in the complexes studied can be arranged in a series of biological efficiency (Scheme 2):

**Table 3:** The results of antibacterial activity against *M. smegmatis*^a^

Compounds	MIC, μg per disk	The zone of inhibition, mm		Ref.
	24 h	24 h	120 h	
1	534	6.6 ± 0.3	6.5 ± 0^b^	This work
2	380	6.5 ± 0.1	0^d^	This work
3	50	7.1 ± 0.3	6.5 ± 0.5^c^	This work
4	21	6.4 ± 0.1	6.1 ± 0^c^	This work
5	12	6.7 ± 0.3	6.6 ± 0.1	This work
[Cu(fur)_2_(phen)]	2	7.0 ± 0.5	7.0 ± 0.5^c^	31
[Fe_3_O(fur)_6_(THF)_3_]·3THF	13	7.0 ± 0.5^c^	0^d^	33
[Zn_2_(fur)_4_(phpy)_2_]	41	6.5 ± 0.5^c^	6.5 ± 0.5^c^	31
[Zn(fur)_2_(bpy)]	44	6.5 ± 0.5^c^	6.5 ± 0.5^c^	32
[Cu(fur)_2_(bpy)(H_2_O)]	46	7.0 ± 0.5	7.0 ± 0.5^b^	32
[Co_3_(fur)_6_(phen)_2_]	60	7.0 ± 0.5	7.0 ± 0.5^b^	33
[Co_3_O(fur)_6_(H_2_O)_3_]	120	6.5 ± 0.3^c^	6.5 ± 0.3^c^	33
[Co_6_(piv)_8_(Hpiv)_4_(fur)_2_(OH)_2_]	143	6.5 ± 0.3^c^	0^d^	33
[Cu_2_(fur)_4_(py)_2_]	146	7.0 ± 0.5	7.0 ± 0.5^b^	31
[Cu(fur)_2_(py)_2_(H_2_O)]	153	7.0 ± 0.5	7.0 ± 0.5^b^	31
[Co(fur)_2_(bpy)]	175	6.5 ± 0.5^c^	6.5 ± 0.5^c^	32
[Cu(fur)_2_(phpy)_2_(H_2_O)]·phpy	224	7.0 ± 0.5	7.0 ± 0.5^b^	34
[Ni(fur)_2_(phen)(H_2_O)_2_]·H_2_O	249	6.7 ± 0.3	6.7 ± 0.3^b^	35
[Zn_2_(fur)_4_(py)_2_]	366	6.5 ± 0.3^c^	6.5 ± 0.3^c^	31
[Cu(fur)_2_(NH_2_-py)_2_]	474	7.0 ± 0.5	7.0 ± 0.5^b^	34
[Ni(fur)_2_(pz)_4_]·2MeCN	635	6.5 ± 0.5	0^d^	35
Hfur	112	0^d^	0^d^	
Bpy	78	0^d^	0^d^	
Neoc	21	6.5 ± 0.06^b^	0^d^	
Phen	8	6.5 ± 0.06^b^	0^d^	
Rif	5	6.5	6.5	
INH	100	7.0^c^	6.5^c^	

a The diameter of the paper disk is 6 mm.

b The zone of inhibition of culture growth does not overgrow within the specified time.

c The zone of inhibition of the growth of the bacterial culture, which initially appeared after several hours of growth, begins to overgrow over the entire surface of the zone.

d 0 – no growth inhibition zone.

Moreover, analysis showed that the inhibition zone has the largest diameter in the case of 3 and does not overgrow during the first day of exposure to the complex, as it was sometimes observed for a number of complexes (Table 3).^31–35^

### Transcriptomic analysis [Cu(fur)_2_(phen)] (RNA-seq)

It is known that copper ions are an active cell death agent. Copper is an important cofactor for various enzymes, however free copper is very toxic to living cells. Copper can damage the structure and functions of enzymes by binding to S-, COO- and NH_2_-containing protein groups. In order to maintain the cellular metabolism at various concentrations of copper in the environment, bacteria have developed certain systems of copper homeostasis that mainly act as defense mechanisms.^51,52^ As with free-living bacteria, protection from copper is of critical importance for the virulence of pathogenic bacteria.

The fast-growing, non-pathogenic *M. smegmatis* species is a model organism for the tuberculosis originator *Mycobacterium tuberculosis*. Analysis of the transcriptome profiles of *M. smegmatis mc*^*2*^*155* strain under the pressure of the most active copper complex [Cu(fur)_2_(phen)] was carried out.^31^

**Scheme 3:**
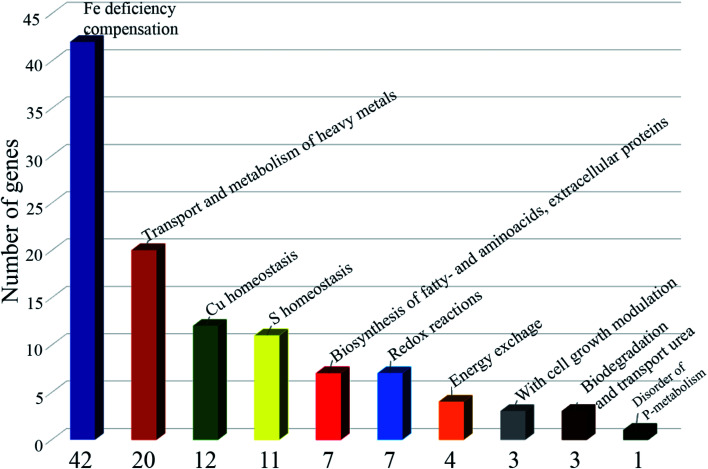
Results of the RNA-seq.

RNA-seq identified 185 differentially expressed genes with parameters: False Discovery Rate (FDR ≤ 0.05; *p* value ≤ 0.05; Fold change FC ≥ 2; log_2_FC ≥ 1): 84 downregulated genes and 101 upregulated genes. 118 (−1.1 > log_2_FC > 1.1) differentially expressed genes were selected for further analysis (Scheme 3). Of the differentially expressed genes, 12 are associated with copper homeostasis, 42 genes are associated with compensation for iron deficiency in the cell; 20 genes, with transport and metabolism of heavy metals, Mo, Ni, Zn, Co, Cd; 11 genes correspond to sulfur homeostasis; 4 genes are associated with energy metabolism; 7 genes are responsible for the biosynthesis of fatty acids, biosynthesis of the cell wall, biosynthesis of amino acids and extracellular proteins; 3 genes are associated with cell growth modulation, 3 genes for biodegradation and transport of urea; 1 gene is expressed when there is a lack of phosphate in the cell; there are 7 genes (−1.9 > log_2_FC > 1.7), functionally annotated, including those associated with redox processes in the cell; in addition, there are 8 genes of hypothetical proteins with unknown functions (1.6 > log_2_FC > 1.6).

Thus, under the pressure of the copper complex, the capture of copper by the cell stops and the outflux of copper from the cell increases. Moreover, violation of homeostasis of microelements vital for the mycobacterium occurs. Cellular processes are initiated to replenish strong iron starvation (iron is an important microelement for *M. smegmatis* and *M. tuberculosis*, as respiration processes are disrupted in case of its deficiency^51^). The absorption of Ni, Zn, Cd, Mo, As and the synthesis of cobalamin (Vit B_12_) decrease; the uptake of sulfur, its transport into the cell and assimilation are hindered; the transport of uric acid from the cell decreases, which may be a consequence of a slowdown in purine metabolism; the synthesis of extracellular proteins, amino/fatty acids and cell wall lipids is modified; hypoxia and phosphate deficiency indicators are activated.

## Conclusions

Thus, complexes of Zn^II^ and Cu^II^ with fur^−^ and Ac^−^ anions incorporating molecules of N-donor ligands (neocuproine and 3-aminopyridine) with polymeric (1) and molecular structures (2–5) have been synthesized. All the complexes are readily soluble in water and show stability according to NMR (^1^H) data upon dissolution in DMSO solutions, as well as in glucose and NaCl solutions according to UV-vis results. The *in vitro* biological activity of the complexes against the model non-pathogenic strain *M. smegmatis* showed the lowest activity of polymeric complex 1. In this case, the transition from Zn^2+^ to Cu^2+^ cations indicate an increase in the activity of copper complexes. RNA-seq for the most active copper complex [Cu(fur)_2_(phen)] made it possible to single out and identify 185 differentially expressed genes, one quarter of which are associated with iron deficiency compensation. The maximum changes in the expression level were recorded in the genes associated with the iron metabolism and transport, transport and metabolism of heavy metals, and copper homeostasis. Under the action of the copper complex, global changes occur in the metabolism and transport of iron and other metals, sulfur, amino acid (in particular arginine and alanine), the biosynthesis of lipids in *M. smegmatis*.

## Conflicts of interest

The authors declare that they have no known competing financial interests or personal relationships that could have appeared to influence the work reported in this paper.

## Supplementary Material

RA-012-D1RA08555G-s001

RA-012-D1RA08555G-s002
