# Supplementary material for: What are the prospects for using complexes of copper(ii) and zinc(ii) to suppress the vital activity of Mycolicibacterium smegmatis?
Source: RSC Adv. 2022 Feb 10;12(9):5173–83. doi: 10.1039/d1ra08555g (PMC8981969; doi:10.1039/d1ra08555g)
Supplement: RA-012-D1RA08555G-s001 [file RA-012-D1RA08555G-s001.pdf]

Electronic Supplementary Material (ESI) for RSC Advances.
This journal is © The Royal Society of Chemistry 2022

Supporting Information

for

What are the prospects for using complexes of copper(II) and zinc(II) to suppress the vital activity of *Mycolicibacterium smegmatis*?

Irina A. Lutsenko[a,*], Dmitry E. Baravikov[a,b], Kseniya A. Koshenskova[a,b], Mikhail A. Kiskin[a], Yulia V. Nelyubina[c], Petr V. Primakov[c], Yu. K. Voronina[a], Veronika V. Garaeva[c,e], Dmytriy A. Aleshin[c], Teimur M. Aliev[c], Valery N. Danilenko[d], Olga B. Bekker[d] and Igor L. Eremenko[a]

[a] *N.S. Kurnakov Institute of General and Inorganic Chemistry of the Russian Academy of Sciences, Leninsky prosp. 31, 119991 GSP-1, Moscow, Russian Federation*
Fax (495) 952 1279. E-mail: irinalu05@rambler.ru

[b] *D. Mendeleev University of Chemical Technology of Russia, Moscow, Russian Federation*

[c] *A.N. Nesmeyanov Institute of Organoelement Compounds, Russian Academy of Sciences, Vavilova Str. 28, 119991 Moscow, Russian Federationc*

[d] *Vavilov Institute of General Genetics, Russian Academy of Sciences, Gubkina, 3,119333 Moscow, Russian Federation*

[e] *Moscow Institute of Physics and Technology, 9 Institutskiy per, Dolgoprudny, Moscow Region, 141701, Russian Federation*

Table S1 Selected crystal data and parameters for structure refinement of the title compounds **1-5**

	**1**	**2**	**3**	**4**	**5**
Empirical formula	$C_{20}H_{12}O_{12}Zn_2$	$C_{30}H_{24}N_4O_{12}Zn_2$	$C_{24}H_{18}N_2ZnO_6$	$C_{18}H_{18}N_2O_4Zn$	$C_{29}H_{24}CuN_2O_{10}$
Formula weight	575.04	763.27	482.81	391.75	624.067
T, K	120	150	120	120	100
Crystal system	monoclinic	triclinic	triclinic	monoclinic	triclinic
Space group	*P2₁/n*	*P*-1	*P*-1	*C2/c*	*P*-1
a (Å)	13.5158(7)	8.3911(4)	9.622(3)	14.561(4)	10.7554(3)
b (Å)	12.8924(6)	9.9264(4)	10.595(3)	9.318(2)	11.1284(3)
c (Å)	13.8937(7)	10.7162(5)	11.815(3)	25.811(6)	12.4298(3)
α (°)	90	63.792(1)	78.455(5)	90	98.575(2)
β (°)	91.1060(10)	74.028(1)	76.399(5)	99.571(15)	107.587(2)
γ (°)	90	73.325(1)	65.951(5)	90	105.477(2)
V(Å^3)	2420.5(2)	755.62(6)	1061.7(5)	3453.27	1323.34(7)
Z	4	1	2	8	2
D_{calc}/ (g·cm^{-3})	1.578	1.677	1.530	1.507	1.566
μ (mm^{-1})	20.40	1.661		14.48	8.91
θ range (°)	52	58	52	54	52
Range of *h, k* and *l*	-16≤h≤16<br>-15≤k≤15<br>-17≤l≤17	-11≤h≤11<br>-13≤k≤13<br>-14≤l≤14	-13≤h≤13<br>-15≤k≤15<br>-16≤l≤16	-20≤h≤20<br>-13≤k≤10<br>-36≤l≤28	-15≤h≤15<br>-15≤k≤15<br>-17≤l≤17
F(000)	1152	388	504	1619	643
Number of parameters	372	315	300	250	403
GOOF	1.054	1.020	0.994	1.0458	1.0591
$R_1(I > 2\sigma(I))$	3.26	2.65	3.65	4.67	5.87
$wR_2(I > 2\sigma(I))$	9.09	6.32	8.81	11.99	13.36
$\Delta\rho_{max}$/ $\Delta\rho_{min}$ (e Å^{-3})	0.601/-0.402	0.48/-0.3	1.121/-0.461	0.532/-0.635	1.5498/-1.7514

Table S2 – Experimental DOSY self-diffusion coefficients of substances for the mixture and complex samples.

Experimantal self-diffusion coefficient, Log(m^2/s) at 305 K	Log($D^{complex}$)	Log(D^{fur})	Log(D^{NEC})	Log(D^{DMSO})
Mixture	-	-9.3141	-9.4074	-9.0343
Complex	-9.5325	-	-	-9.0318

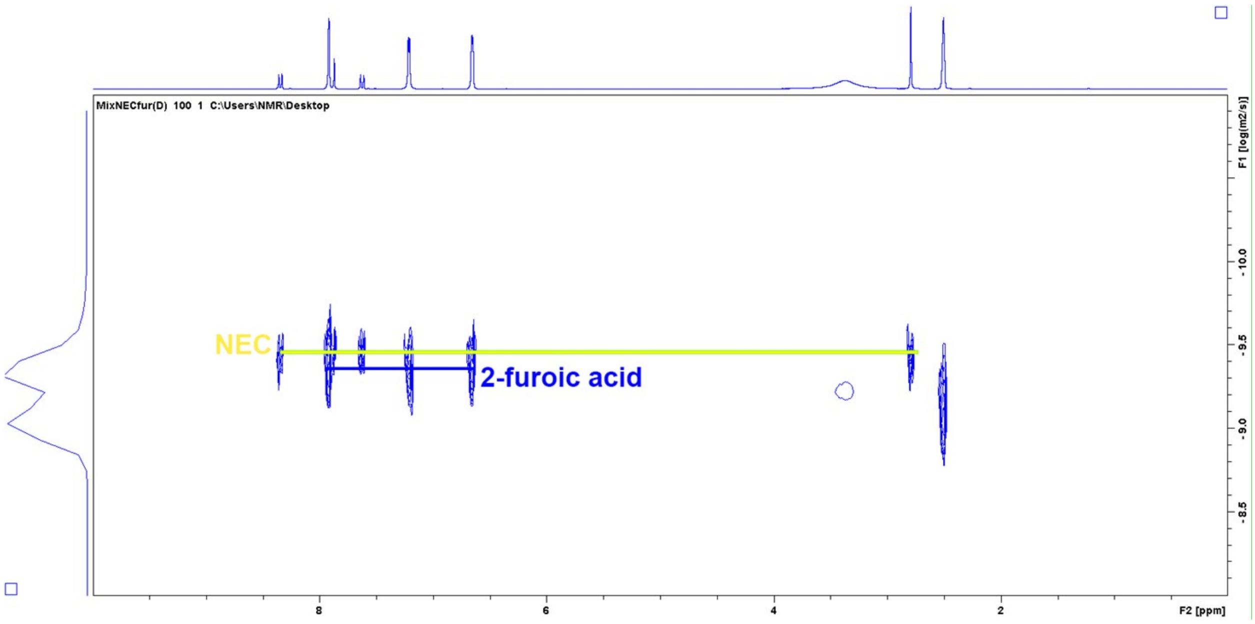

Fig. 1S. 2D DOSY spectrum of mixture neoc and 2-Hfur.

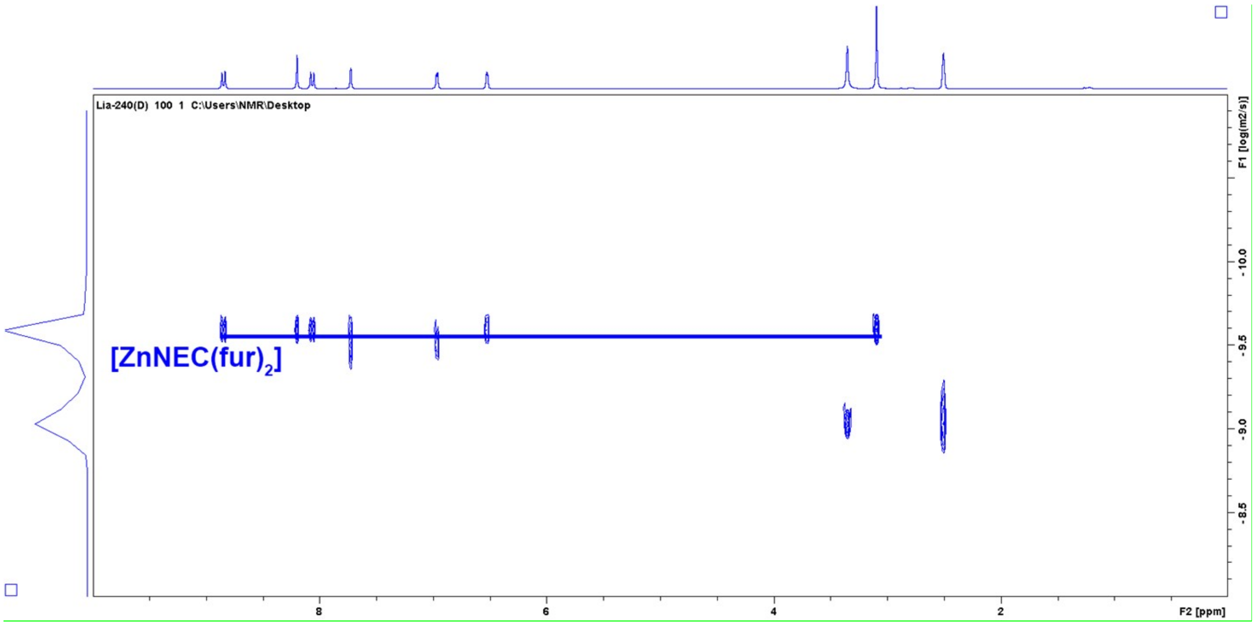

Fig. 2S. 2D DOSY spectrum of the complex **3**.

Table. S3. Acquisition parameters for DOSY experiments.

Parameters	P1, μs	δ (small delta), μs	Δ (big delta), s	D1, s	NS
Mixture	12.6	1000	0.2	3	16
Complex	11.5	1000	0.2	3	16
